# Perioperative Blood Loss Can Be Reduced If Total Knee Arthroplasty Was Performed in the Si Hour-Period, Compared with the Wei Hour-Period: A Retrospective Cohort Study

**DOI:** 10.1155/2021/9990413

**Published:** 2021-08-20

**Authors:** Xiaojian Wang, Ting Xu, Rui Wang, Penghe Wang, Shuaijie Jin, Peijian Tong, Shuaijie Lv

**Affiliations:** ^1^The First Clinical College of Zhejiang Chinese Medical University, Hangzhou, Zhejiang, China; ^2^The Third Clinical College of Zhejiang Chinese Medical University, Hangzhou, Zhejiang, China; ^3^The First Affiliated Hospital of Zhejiang Chinese Medical University, Hangzhou, Zhejiang, China

## Abstract

**Objective:**

To investigate the efficacy and safety of performing primary unilateral total knee arthroplasty (TKA) in the “Si hour-period” meaning 09:00 a.m. to 11:00 a.m. (one of the 12 two-hour periods into which the day was traditionally divided, each being given the name of one of the 12 earthly branches), compared with the “Wei hour-period” (13:00–15:00).

**Methods:**

Patient documentations were studied for those who underwent a primary unilateral TKA performed by the same surgical team with a tourniquet between January 2018 and January 2021 at our medical center. Eighty-four patients were enrolled and assigned into group A (in Si hour-period) and group B (in Wei hour-period). The main outcomes were total blood cell loss (TBL), hidden blood loss (HBL), visible blood loss (VBL), maximum hemoglobin (Hb) drop, and transfusion rate. Secondary outcomes were length of hospital stay (LOS), postoperative femorotibial mechanical axis (FTMA), FTMA correction, platelet count, plasma D-dimer (D-D), prothrombin time (PT), international normalized ratio (INR), and the incidence of postoperative complications.

**Results:**

Group A showed statistical significance lower at the mean TBL, the mean HBL, and the maximum Hb drop (95% *CI*: −352.8 to −46.1,*P*=0.011, 95% *CI:* −348.0 to −40.1,*P*=0.014, and 95% *CI:* −9.5 to −0.7,*P*=0.023, respectively) after TKA than group B. The postoperative platelet count of group A was more significant than that of group B (95% *CI*:3.1 to 52.9, *P*=0.028). The VBL, transfusion rate, the LOS, postoperative FTMA, FTMA correction, plasma D-D, PT, INR, and the incidence of postoperative complications (wound complications, calf muscular vein thrombosis, infection, pulmonary embolism, and deep vein thrombosis) were similar between the two groups (*P* > 0.05, respectively).

**Conclusion:**

Our study shows that blood loss can be reduced when TKA is performed in the “Si hour-period,” which may be due to increasing platelet count, and postoperative complications did not increase, compared with the Wei hour-period. We recommend that the selective operation, such as TKA, should be performed in the “Si hour-period” in clinical practice between the two hour-period.

## 1. Introduction

Knee osteoarthritis (KOA) is a chronic bone and joint disease and related to age. Its prevalence is increasing year by year due to the continuous progress of population aging [1]. The data [2] showed the proportion of doctor-diagnosed KOA is estimated to be 15.7% in the population aged ≥45 by the year 2032. Total knee arthroplasty (TKA) is an effective and well-established procedure for the treatment of advanced KOA. However, it can cause postoperative bleeding and even severe anemia for surgical trauma and fibrinolysis [3, 4]. The common methods for reducing perioperative bleeding are the application of tranexamic acid (TXA), tourniquet, and postoperative compression. In actual clinical practice, however, it is found that blood loss is not effectively controlled as expected. Also, these methods are controversial due to the postoperative complications [5–8]. Therefore, as orthopedics doctors, we need to explore ways to reduce bleeding for a more optimized perioperative management of TKA.

The “biological clock” theory means that mammals regulate the circadian rhythm of cells through the suprachiasmatic nucleus at the base of the hypothalamus, which causes the body's physiological activities to also show rhythmic changes [9, 10]. Modern medicine combines the biological clock theory with disease activity to form the chronomedicine theory [11–13]. The chronomedicine in traditional Chinese medicine (TCM) is mainly based on the concept of “correspondence between man and universe,” using TCM basic theories to analyze the correlation between human physiology, pathology, and rhythm, to guide the prevention and treatment of diseases in clinical practice [14]. Many studies [15–17] have shown that the outcome of the treatment group based on chronomedicine intervention is better than that of the control group. So, these brought us a new consideration: can the TKA operation hour-period affect the perioperative bleeding and complications? Therefore, we conducted a retrospective cohort study to investigate the correlation between the operation time of TKA and blood loss, coagulation, length of hospital stay (LOS), femorotibial mechanical axis (FTMA), namely, the hip-knee-ankle angle (HKA), and postoperative complications.

## 2. Materials and Methods

### 2.1. Study Design

This was a retrospective cohort study. We performed this study in the Department of Orthopedics, the First Affiliated Hospital of Zhejiang Traditional Chinese Medicine University. All patients who underwent primary unilateral single-stage TKA from January 2018 to January 2021 were included.

Inclusion criteria were complete medical records, unilateral primary TKA, according with the diagnostic criteria of KOA and in Kellgren–Lawrence [18] grade IV, normal preoperative test results, and volunteering to participate in this study and signed the informed consent form.

Exclusion criteria were previous history of knee surgery, taking nonsteroidal anti-inflammatory drugs (NSAIDs) and other drugs that may affect blood coagulation in the past 6 months, hematological or rheumatologic osteoarthritis etiologies, history of deep vein thrombosis (DVT), active phlebitis, a coagulation disorder, untreated diabetes, and untreated hypertension.

Eighty-four patients (26 males, 58 females) were included and assigned to two groups: group A (*n* = 43) where the operation time was “Si hour-period” (09:00 a.m. to 11:00 a.m., one of the 12 two-hour periods into which the day was traditionally divided, each being given the name of one of the 12 earthly branches) and group B (*n* = 41) where the operation time was “Wei hour-period” (13:00 to 15:00).

### 2.2. Arthroplasty Procedures

For perioperative prophylaxis, cefazolin sodium antibiotics were administered 30 min before surgery and 24 h after surgery. General anesthesia was performed in all patients. The arthroplasty procedures were carried out by the same group of senior surgeons, using midline anterior incision with a medial parapatellar approach and with the use of posterior cruciate-stabilizing knee prosthesis (Stryker^®^, NGR). The pneumatic tourniquet was inflated before the incision and deflated after the closure of the incision. The inflation time and amount of visible blood loss (VBL) intraoperatively were recorded. Local injection of 20 ml 5% TXA into periarticular soft tissue was performed to decrease bleeding before closing the incision. There was no drainage in any patient. The postoperative analgesia protocols and anticoagulant therapy, the incision care, and rehabilitation program were standardized and similar in the two groups. Postoperatively, the elastic bandage and patient-controlled analgesia pump were applied for 24 h and 48 h, respectively, and anticoagulant therapy was initiated 6 hours in all patients [19]. All patients received the same rehabilitation program.

### 2.3. Outcome Measurement Details

#### 2.3.1. Blood Loss

Blood loss included total blood loss (TBL), VBL, and hidden blood loss (HBL). The TBL is the sum of the VBL during the operation, the HBL postoperatively, and transfusion. The preoperative patient blood volume (PBV) and TBL were estimated by Nadler's formula [20] and the Gross formula [21], respectively.

The preoperative PBV = *K*1 × height^3^ (m^3^) + *K*2 × weight (kg) + K3. Male: *K*1 = 0.3669, *K*2 = 0.03219, and *K*3 = 0.6041. Female: *K*1 = 0.3561, *K*2 = 0.03308, and *K*3 = 0.1833.

The TBL = the preoperative PBV × (Hcit1 – Hct2)/Hct3. Hct1 means initial preoperative hematocrit (Hct) level. Hct2 means the lowest Hct postoperative. Hct3 means the average of Hct1 and Hct2. The VBL was reckoned by the amount of the sum of liquid in the gauze and in the negative pressure drainage bottle minus the amount of saline. If the patient was treated with blood transfusion, the blood transfusion volume should be included in the TBL. One unit of concentrated red blood cell is equivalent to 200 ml of standard red blood cell volume. Blood transfusion depends on the postoperative Hb value of patients, which should be considered if the postoperative Hb value is less than 70 g/L in patients or between 70 and 100 g/L in symptomatic patients [22].

#### 2.3.2. The Maximum Hemoglobin (Hb) Drop

The maximum Hb drop was calculated by the difference between the level of Hb preoperatively and the lowest level during the postoperative hospital stay.

#### 2.3.3. Length of Hospital Stay (LOS)

The length of hospital stay was considered as days from the date of admission to discharge.

#### 2.3.4. Limb Alignment on Mechanical Axis (MA) Radiographs

The alignment of the lower limbs is represented by the FTMA, namely, HKA, which reflects the deformity of the legs. Preoperative and postoperative HKA were calculated twice by one author (PH) and measured independently twice by two authors (TX, SJ) to increase reliability.

#### 2.3.5. FTMA Correction

Limb alignment was classified as neutral (HKA = 180° ± 3°), varus (HKA < 177°), or valgus (HKA > 183°) [23]. We tried to ensure that the HKA was corrected to neutral in intraoperative. The FTMA correction was calculated by the difference between the preoperative and postoperative HKA, which will affect the intraoperative osteotomy procedure that is related to the amount of bleeding.

#### 2.3.6. Coagulation

Perioperative blood routine examination, plasma D-dimer (D-D), coagulation action test, and renal function were measured preoperatively and on postoperative days (POD) 1, 3, 7, and 14.

#### 2.3.7. Complications within POD-30

Complications were recorded during hospitalization and POD-30 through the outpatient system. (1) Incision complications: poor incision healing, peripheral redness and swelling, infection, etc; (2) thrombotic complications: the lower extremity intramuscular VT, DVT, and pulmonary embolism (PE). Venous ultrasonography or pulmonary computed tomography angiography (CTA) was performed when DVT or PE was suspected.

### 2.4. Ethical Considerations

This study was approved by the ethics committee of the First Affiliated Hospital of Zhejiang Chinese Medical University. All participants signed informed consent, were free to withdraw anytime, and well informed about this study purpose, methods, and procedures.

### 2.5. Statistical Analysis

All data analyses were performed using SPSS version 25.0 (IBM, Armonk, NY, USA). Mean ± standard deviation or median (IQR) or frequency (percentage) was used to summarize variables as appropriate. Normality of data was tested by the Shapiro–Wilk test. The continuous variable differences between two groups were calculated using Student's *t*-test or the Wilcoxon–Mann–Whitney test. The chi-square test (*χ*^2^) or continuity correction test were used for the comparisons of categorical variables between groups. A *P* value <0.05 was considered statistically significant.

## 3. Results

### 3.1. Patient Baseline Characteristics

Eighty-four patients were eligible for analysis, between January 2018 and January 2021. There were no dropouts or withdrawals. The demographic details, preoperative variables, and operation time were homogenous and comparable between the two groups (Table 1).

### 3.2. The Primary Outcomes

It shows significant difference in the TBL, HBL, and the maximum Hb drop by Student's *t*-test. The mean difference of TBL, HBL, and the maximum Hb drop were −199.4 ml (95% *CI:* −352.8 to −46.1,*P*=0.011), −194.1 ml (95% *CI:* −348.0 to −40.1,*P*=0.014), and −5.1 g/L (95% *CI:* −9.5 to −0.7,*P*=0.023), respectively, between groups A and B. Student's *t*-test and the chi-square test show no significant difference in VBL and blood transfusion rate (*P*=0.314 and *P*=0.956, respectively; Table 2).

### 3.3. The Secondary Outcomes

No significant difference was identified in LOS, post-HKA, FTMA correction, plasma D-D, prothrombin time(PT), and international normalized ratio(INR) between the two groups (*P* > 0.05, respectively). However, the postoperative platelet count is significantly higher than that in group B (95% *CI:* 3.1 to 52.9,*P*=0.028; Table 2). The postoperative level of plasma D-D, PT, INR, and platelets count was higher than the preoperative. The most post-HKA was in the neutral.

### 3.4. Complications

The main postoperative complications of the two groups were incision delayed healing, incision of redness and swelling, and intramuscular vein thrombosis (VT), which has no statistically significant difference (*P* > 0.05, respectively; Table 3). There was no infection, DVT, PE, and other adverse events in the two groups.

## 4. Discussion

TKA has been more and more widely used for it can effectively relieve the pain of severe KOA and improve the joint function, with the gradual improvement of people's quality of life, the continuous improvement of prosthesis design, and the continuous improvement of the operator's operation technology [24]. However, its development has been affected by the postoperative anemia and complications caused by excessive blood loss and which reduced the satisfaction of patients in clinical practice [1, 25]. Although allogeneic blood transfusion can replenish excessive blood loss and its safety has been greatly improved in recent years, it could still lead to prolonged LOS, increased medical costs, and a variety of complications, such as immune response, and infection, which is not conducive to the rehabilitation of patients and brings a certain economic burden to the society [25–27]. Therefore, reducing perioperative blood loss is one of the keys of enhanced recovery after surgery.

Our study has shown that better outcomes could be received interventions based on the chronomedicine of TCM, of which the representative is the application of the theory of midnight-noon ebb-flow in clinical practice, which mainly refers to the ups and down rhythm of the flow of Qi and blood through the twelve meridians [28]. The theory suggests that the Qi and blood of the human body flow to different viscera through the corresponding meridians in different hour periods in orderly, circularly, and the beginning connect with end way, which brings about the difference of the physiological functions of the viscus [28]. The traditional 12 two-hour periods were the twelve-hour periods dominated by the twelve viscera. The hour-period and the corresponding viscus are listed as follows: Zi hour-period (23:00–01:00), Chou hour-period (01:00–03:00), Yin hour-period (03:00–05:00), Mao hour-period (05:00–07:00), Chen hour-period (07:00–09:00), Si hour-period (09:00–11:00), Wu hour-period (11:00–13:00), Wei hour-period (13:00–15:00), Shen hour-period (15:00–17:00), You hour-period (17:00–19:00), Xu hour-period (19:00–21:00), and Hai hour-period (21:00–23:00) correspond to the Gallbladder Meridian, the Liver Meridian, the Lung Meridian, the Large Intestine Meridian, the Stomach Meridian, the Spleen Meridian, the Heart Meridian, the Small Intestine Meridian, the Bladder Meridian, the Kidney Meridian, the Pericardium Meridian, and the Triple Energizer Meridian, respectively. According to the theory of TCM that “the spleen controls blood” and “the liver stores blood,” the spleen and the liver are viscera that can prevent bleeding, and its controlling time is Si hour-period and Chou hour-period, respectively. In considering the reality that the operation was performed more in the morning and afternoon in our medical center, we studied the correlation between the operation hour-period and the amount of bleeding and complications.

A finding in our analysis was that the TBL, HBL, and the maximum Hb drop were less in group A compared with those in group B. This indicates that the intervention guided by the chronomedicine theory of TCM is effective, which lines with others literature [29]. In terms of intraoperative blood loss, there was no significant difference between two groups. We surmised that the intraoperative bleeding has been well abated for the measure of modern medicine; for example, the pneumatic tourniquet was applied during the operation. At present, the use of tourniquet in TKA is the subject of ongoing controversial discussion with no clear agreement about the advantages and disadvantages of it. It is well accredited that the use of it reduces intraoperative blood loss [30, 31] and optimizes visualisation, thereby shortening surgical duration [32, 33]. Zak et al. [34] regarded that its utilization has minimal impact on postoperative pain scores and opioid consumption, compared without a tourniquet. The disadvantages of it mainly include damaging blood vessels and local soft tissue, increasing fibrinolytic activity [35] and postoperative HBL [36], leading to local tissue swelling or hypoxia, which affects wound healing [37, 38], decreasing muscle strength [39], and limiting range of motion [40]. However, there are no unified understanding of these issues. Before the academic consensus, our center is still using tourniquet in full course of all TKA. Therefore, we can exclude the influence of the tourniquet, as a powerful variable in this study. In addition, this study provided evidence for the safety of performing in Si hour-period, on account of insignificant differences in D-D, PT, and rate of complications between the two groups.

In light of the results of this study, we supposed the following several reasons. To begin with, the function of the TCM spleen is stronger in the Si hour-period that the spleen is in charge. The stimulation of the incision (in the area between the Spleen Meridian and the Stomach Meridian) could promote the Qi and Blood flow of the Spleen Meridian, which further enhanced the controlling blood function of the spleen. By the way, the Stomach Meridian and the Spleen Meridian interact with each other in the TCM theory. Afterwards, the TCM spleen can reduce bleeding by increasing the number of platelets probably. Moreover, the patients do not need to wait in the ward for a long time, which brings about a better physical and psychological state for both patients and doctors, as the Si hour-period is generally the first operation period. These opinions need to be demonstrated by high-quality research.

Our study is the first to explore the relationship between the operation hour-period with blood loss and complications, which is of significance to the field of TKA. There were some limitations in our study. Firstly, we cannot exclude the possible reason that the differences between the two periods would be the concentration or fatigue of the surgeons at the different times. TKA will be the first operation in our group in a day, and the team will have a short break if the operation was scheduled in the afternoon, whereas the biased results of the study were due to the psychological and physical consumption caused by outpatient service or other work in the morning, excluding surgery, which cannot be ruled out. We plan to design another high-quality study to get better and more accurate results. Moreover, this current study is not a randomized controlled trial. The retrospective nature of this study makes it more susceptible to selection bias and confounding. Also, this is a single-center study, and its results may not be applicable to all centers. Afterwards, patients underwent ultrasound and CT tests only before surgery, and we did not conduct an ultrasound or CTA scan for each patient to assess the presence of DVT or PE after surgery, which caused some asymptomatic patients to be missed. Eventually, this study compared the two hour-period with a small sample size. A large sample size study should be put up to compare the results of other hour-period of TKA in order to determine the best.

## 5. Conclusions

Our study shows that blood loss can be reduced when TKA is performed in the “Si hour-period,” which may be due to increasing platelet count, and postoperative complications did not increase, compared with the Wei hour-period. We recommend that the selective operation, such as TKA, should be performed in the “Si hour-period” in clinical practice in the two hour-period.

## Figures and Tables

**Table 1:** Comparison of baseline characteristics and clinical features between the 2 groups.

Variable	Group A (*n* = 43)	Group B (*n* = 41)	*P* value
Male (*n,* %)	12/26.7%	14/33.3%	0.497^*∗*^
Age (years)	67.4 ± 7.5	68.8 ± 7.7	0.421^*∗∗*^
BMI (kg/m^2^)	25.7 ± 2.9	24.4 ± 3.5	0.079^*∗∗*^
ASA (I/II/III, *n*)	3/33/7	3/31/7	0.993^*∗*^
Operation of left side (*n*, %)	21/48.8%	16/39.0%	0.310^*∗*^

*Comorbidity*			
Hypertension (*n,* %)	11/25.6%	10/24.4%	0.900^*∗*^
Diabetes (*n,* %)	1/2.3%	3/7.3%	0.575^*∗*^
Coronary heart disease (*n,* %)	0	2/4.9%	0.453^*∗*^
Operation time (min)	94.9 ± 12.4	97.7 ± 11.3	0.284^*∗∗*^

*Pre-FTMA*			
Minimum∼maximum(°)	165.5∼195.7	163.4∼197.8	
HKA (°)	173.6 (3.9)	175.6 (6.6)	0.110^*∗∗∗*^

*Preoperative test*			
Hemoglobin (g/L)	130.9 ± 10.2	134.5 ± 12.3	0.146^*∗∗*^
Hematocrit (%)	39.8 ± 2.4	39.9 ± 3.3	0.833^*∗∗*^
Platelet count (^*∗*^10^9^/L)	195.8 ± 38.4	189.9 ± 45.1	0.518^*∗*^
D-D (mg/L)	0.35 (0.35)	0.31 (0.29)	0.588^*∗∗∗*^
PT (s)	11.1 (0.70)	11.1 (0.70)	0.767^*∗∗∗*^
INR	0.91 (0.08)	0.90 (0.08)	0.654^*∗∗∗*^

BMI, body mass index; ASA, American Society of Anesthesiologists; Pre-FTMA, preoperative femorotibial mechanical axis; HKA, hip-knee-ankle angle; D-D, D-dimer; PT, prothrombin time; INR, International Normalized Ratio. ^*∗*^*χ*^2^ test; ^*∗∗*^Student's *t* test; ^*∗∗∗*^Wilcoxon–Mann–Whitney test.

**Table 2:** Comparison of blood loss, maximum Hb drop, transfusion, postoperative test and radiography, and LOS between the two groups.

Variable	Group A (*n* = 43)	Group B (*n* = 41)	Difference (95% CI)	*P* value
TBL (ml)	953.2 ± 294.7	1152.6 ± 405.6	−199.4 (−352.8 to −46.1)	**0.011** ^*∗*^
HBL (ml)	871.1 ± 292.2	1065.1 ± 410.0	−194.1 (−348.0 to −40.1)	**0.014** ^*∗*^
VBL (ml)	82.1 ± 22.8	87.5 ± 25.7	−5.4 (−15.9 to 5.2)	0.314^*∗*^
Maximum Hb drop (g/L)	27.5 ± 11.0	32.6 ± 8.9	−5.1 (−9.5 to −0.7)	**0.023** ^*∗*^
Transfusion (*n,* %)	2/4.7%	3/7.3%	—	0.956^*∗∗*^
LOS (days)	13.9 ± 5.9	13.9 ± 5.9	—	0.856^*∗*^
Post-HKA(°)	179.2 (1.2)	179.4 (1.2)	—	0.363^*∗∗∗*^
FTMA correction(°)	6.1 (3.2)	5.0 (5.5)	—	0.102^*∗∗∗*^

*Postoperative test*				
Platelet count (^*∗*^10^9^/L)	238.1 ± 48.4	210.2 ± 64.4	28.0 (3.1 to 52.9)	**0.028** ^*∗*^
D-D (mg/L)	5.6 ± 2.0	5.0 ± 2.4	0.7 (−0.3 to 1.6)	0.181^*∗*^
PT (s)	11.5 (0.80)	11.6 (1.30)	—	0.771^*∗∗∗*^
INR	0.95 (0.08)	0.95 (0.12)	—	0.854^*∗∗∗*^

TBL, total blood loss; HBL, hidden blood loss; VBL, visible blood loss; Hb, hemoglobin; post-HKA, postoperative hip-knee-ankle angle; FTMA, femorotibial mechanical axis; D-D, D-dimer; PT, prothrombin time; INR, International Normalized Ratio. ^*∗*^Student's *t* test; ^*∗∗*^*χ*^2^ test; ^*∗∗∗*^Wilcoxon–Mann–Whitney test. Boldface indicates *P* value <0.05.

**Table 3:** Complications during POD-30 between the two groups.

Variable	Group A (*n* = 43)	Group B (*n* = 41)	*P* value
Incision delayed healing	1/2.3%	1/2.4%	1.000^*∗*^
Incision of redness and swelling	1/2.3%	2/4.9%	0.966^*∗*^
Infection	0	0	N/A
Intramuscular VT	3/7.0%	2/4.9%	1.000^*∗*^
DVT	0	0	N/A
PE	0	0	N/A
Shock	0	0	N/A
Acute renal failure	0	0	N/A

POD, postoperative days; VT, venous thrombosis; DVT, deep vein thrombosis; PE, pulmonary embolism. ^*∗*^*χ*^2^ test; N/A, not available.

## Data Availability

The datasets used and analysed by this study are available from the corresponding author on reasonable request.

## Abbreviations

KOA: Knee osteoarthritis
TKA: Total knee arthroplasty
BMI: Body mass index
TXA: Tranexamic acid
FTMA: Femorotibial mechanical axis
HKA: Hip-knee-ankle angle
VT: Venous thrombosis
DVT: Deep vein thrombosis
PE: Pulmonary embolism
ASA: American Society of Anesthesiologists
TCM: Traditional Chinese medicine
LOS: Length of stay
VBL: Visible blood loss
TBL: Total blood loss
HBL: Hidden blood loss
PBV: Patient blood volume
Hct: Hematocrit
Hb: Hemoglobin
D-D: D-dimer
POD: Postoperative days
CTA: Computed tomography angiography
PT: Prothrombin time
INR: International Normalized Ratio.
